# Supplementary material for: Stability Enhancement of Freeze-Dried Gelatin/Alginate Coacervates for bFGF Delivery
Source: Pharmaceutics. 2022 Nov 22;14(12):2548. doi: 10.3390/pharmaceutics14122548 (PMC9781200; doi:10.3390/pharmaceutics14122548)
Supplement: Supplementary file 1 [file pharmaceutics-14-02548-s001.zip › pharmaceutics-2018590-supplementary.pdf]

Supporting Information
Fig. S1

Thermal analysis

(a)

TGA of high molecular weight gelatin (HWGA)

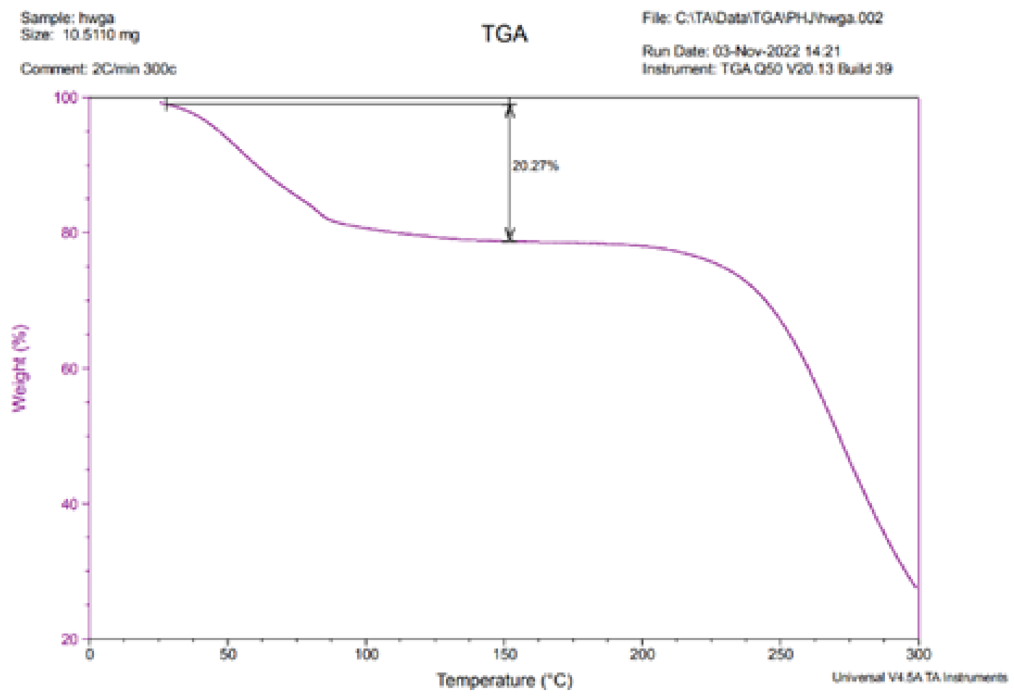

TGA of sodium alginate (SA)

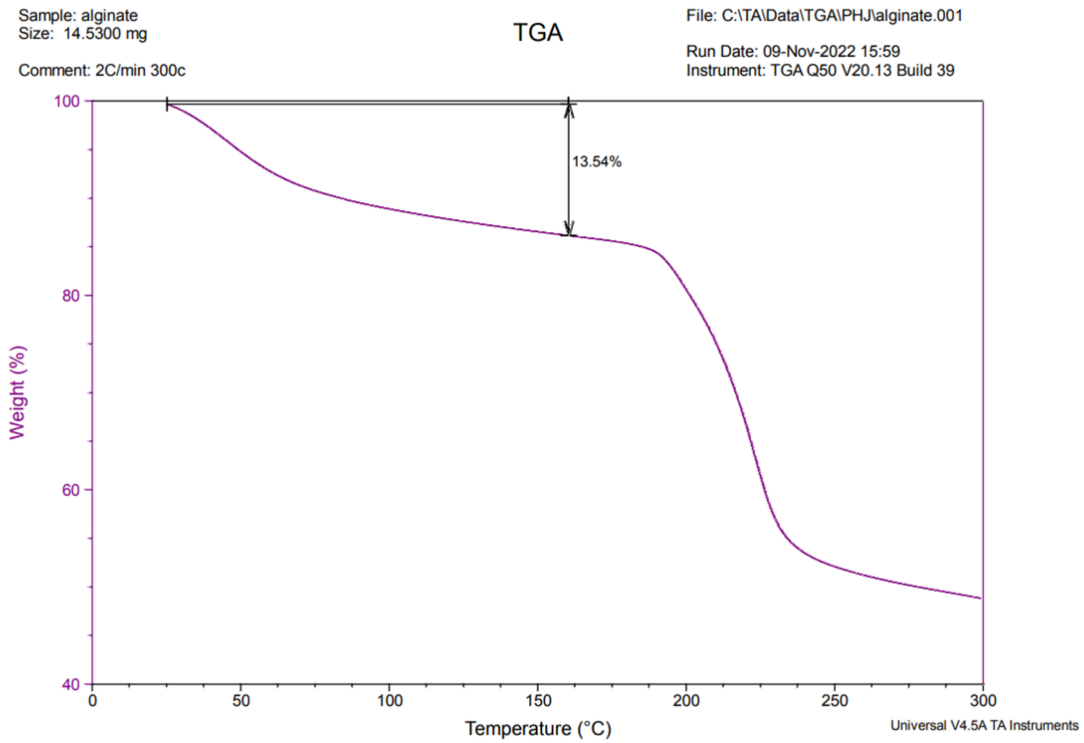

(b)

TGA of freeze-dried GA/SA coacervate without trehalose

TGA of freeze-dried GA/SA coacervate with trehalose

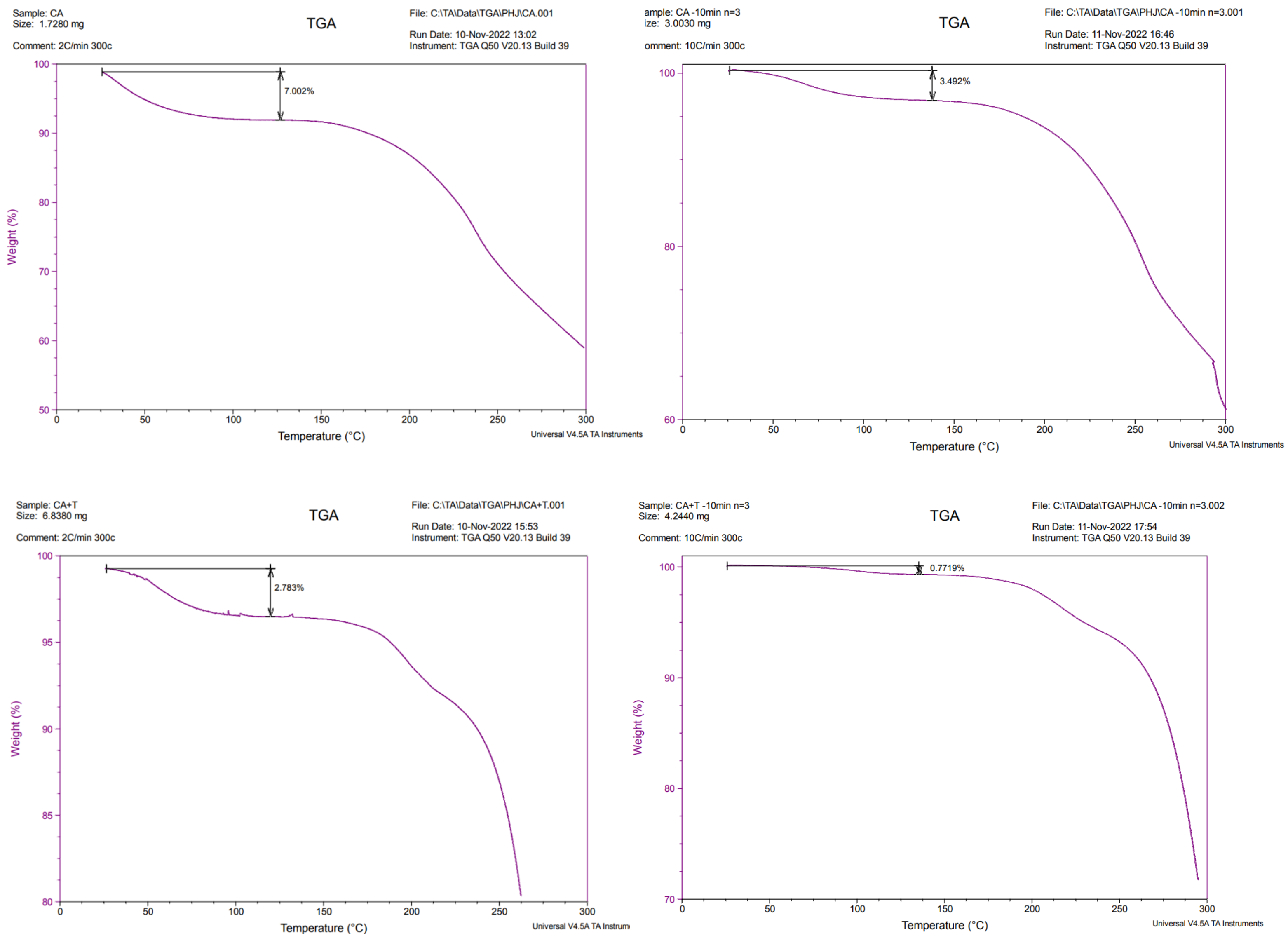

(c)

DSC of high molecular weight gelatin (HWGA)

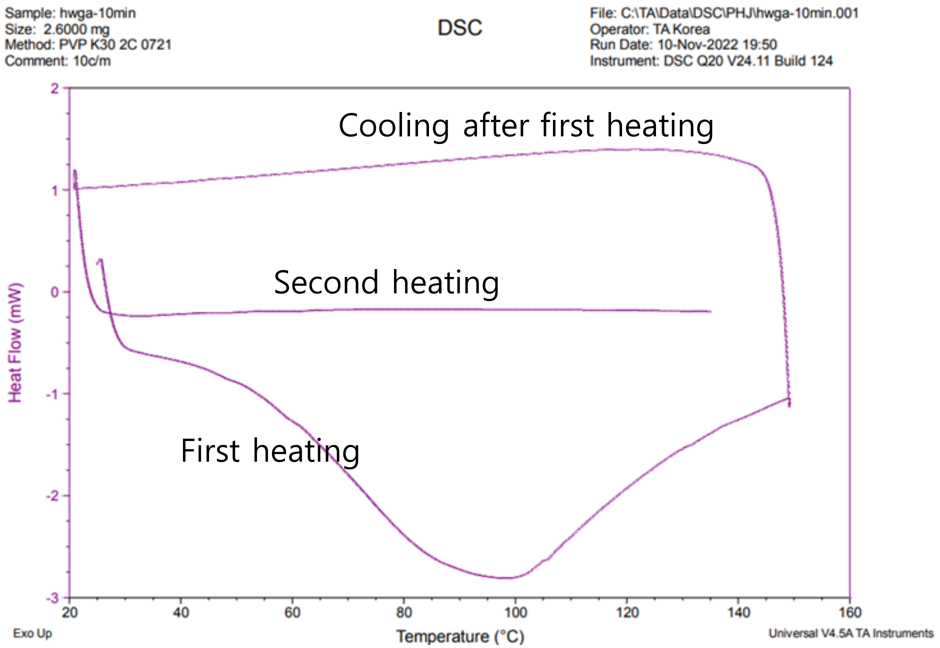

DSC of sodium alginate (SA)

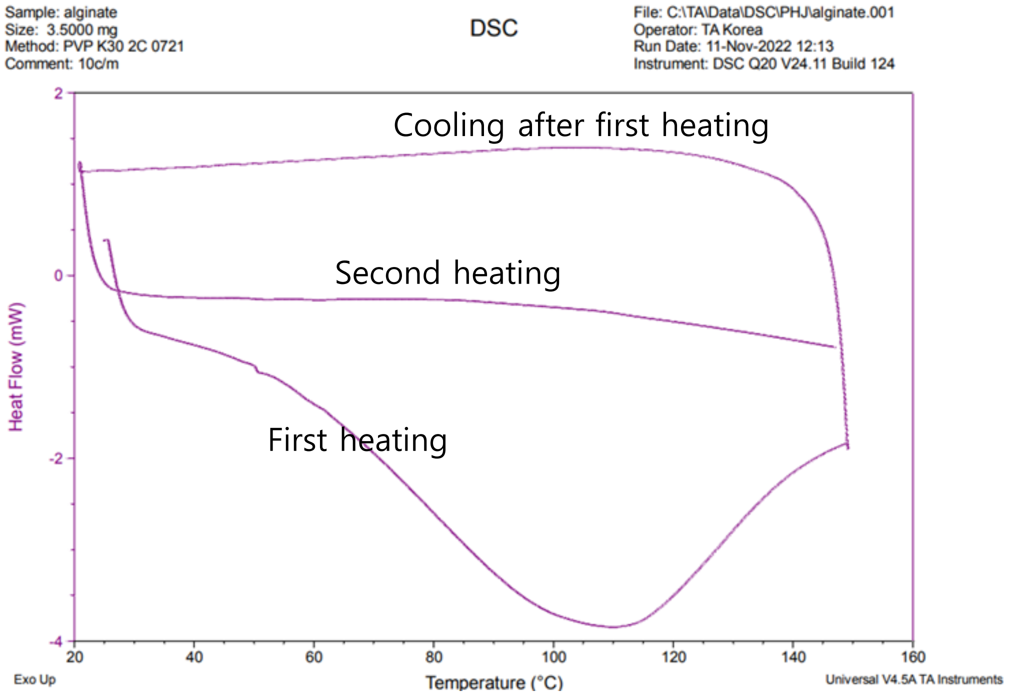

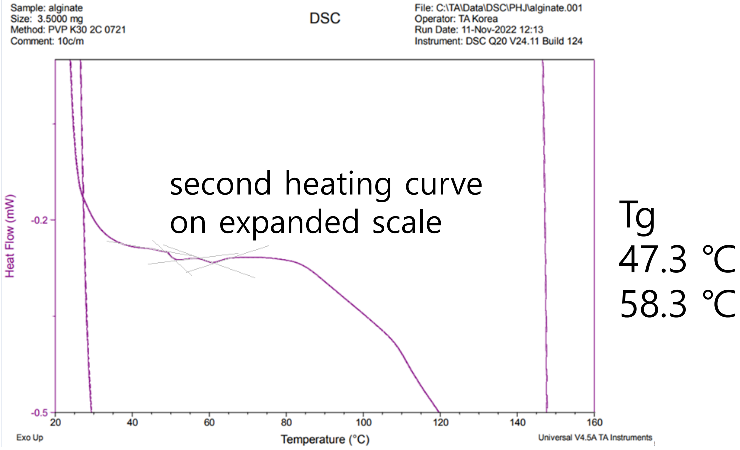

Tg
47.3 ℃
58.3 ℃

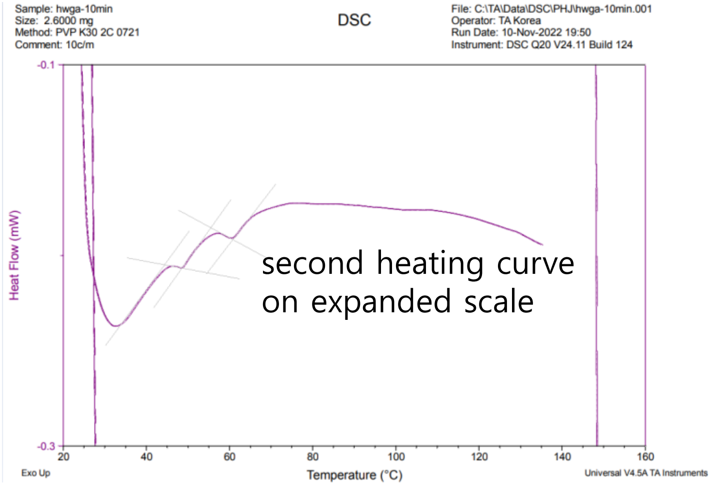

Tg
49.7 ℃
58.1 ℃

(d) heating-cooling-heating

DSC of freeze-dried GA/SA coacervate without trehalose

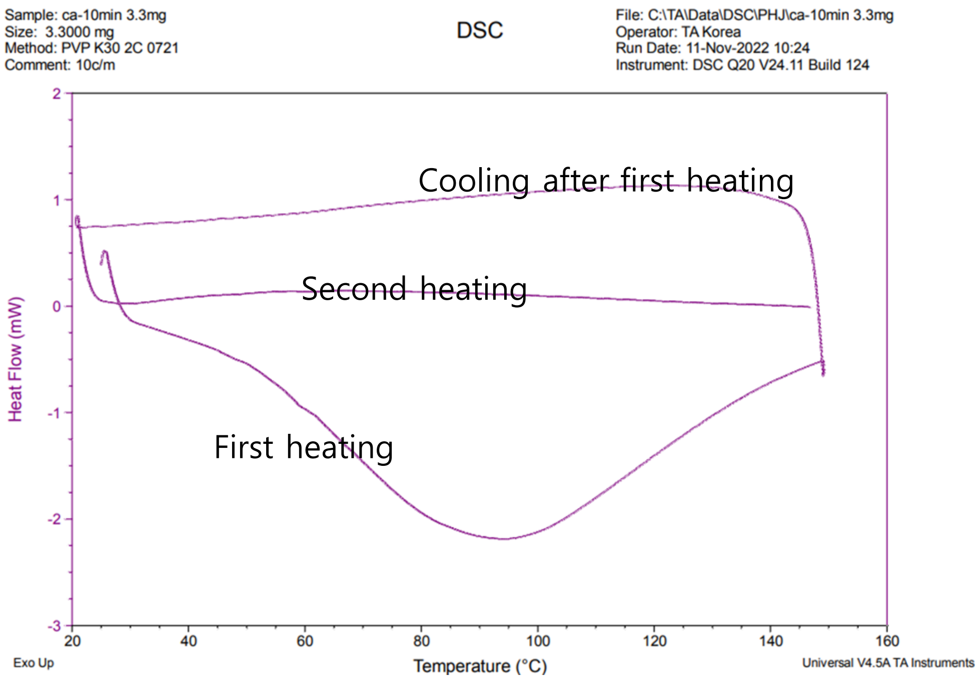

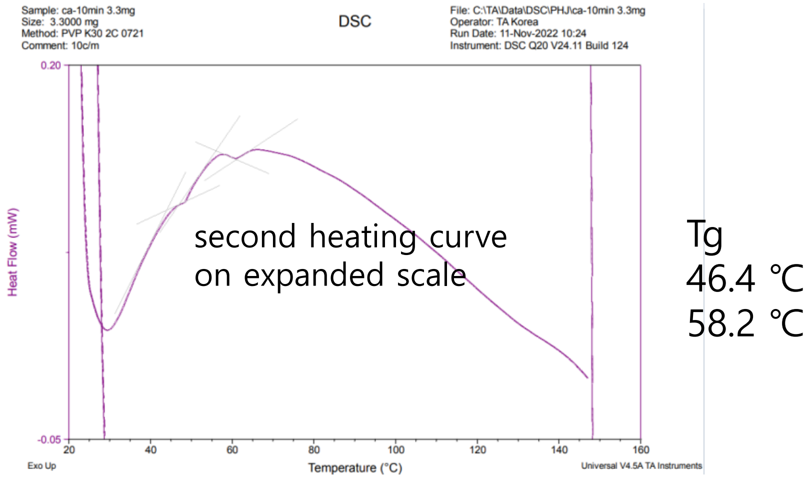

Tg
46.4 ℃
58.2 ℃

DSC of freeze-dried GA/SA coacervate with trehalose

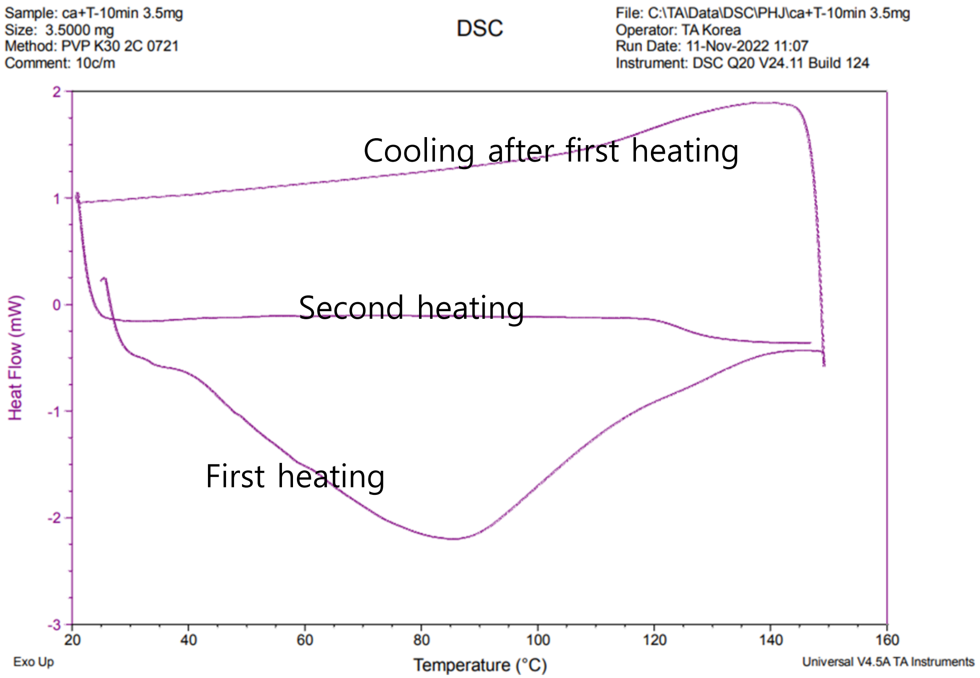

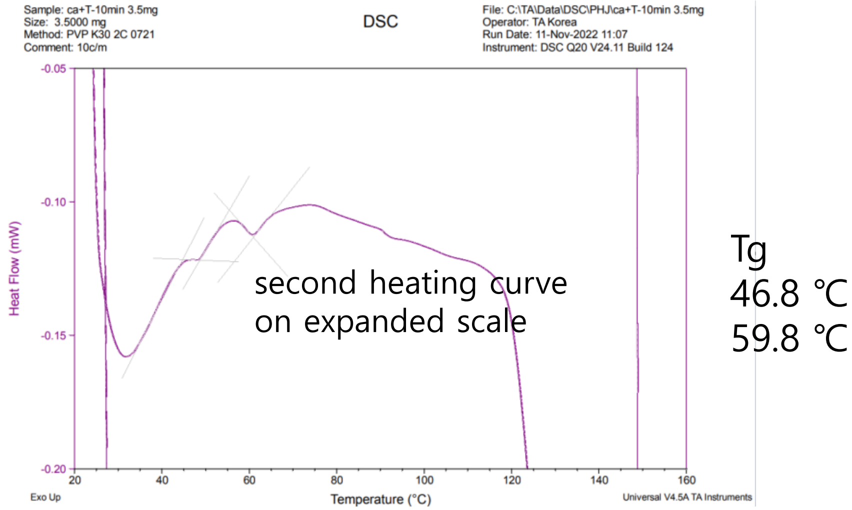

Tg
46.8 ℃
59.8 ℃

(d) heating-10 min stay at 100 °C-cooling-heating

DSC of freeze-dried GA/SA coacervate without trehalose

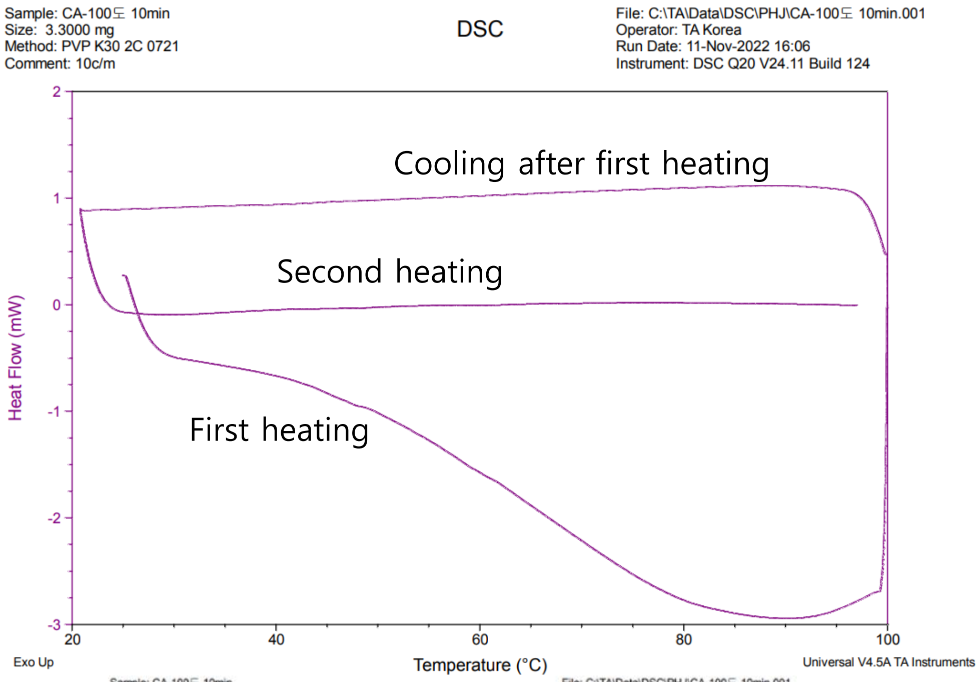

DSC of freeze-dried GA/SA coacervate with trehalose

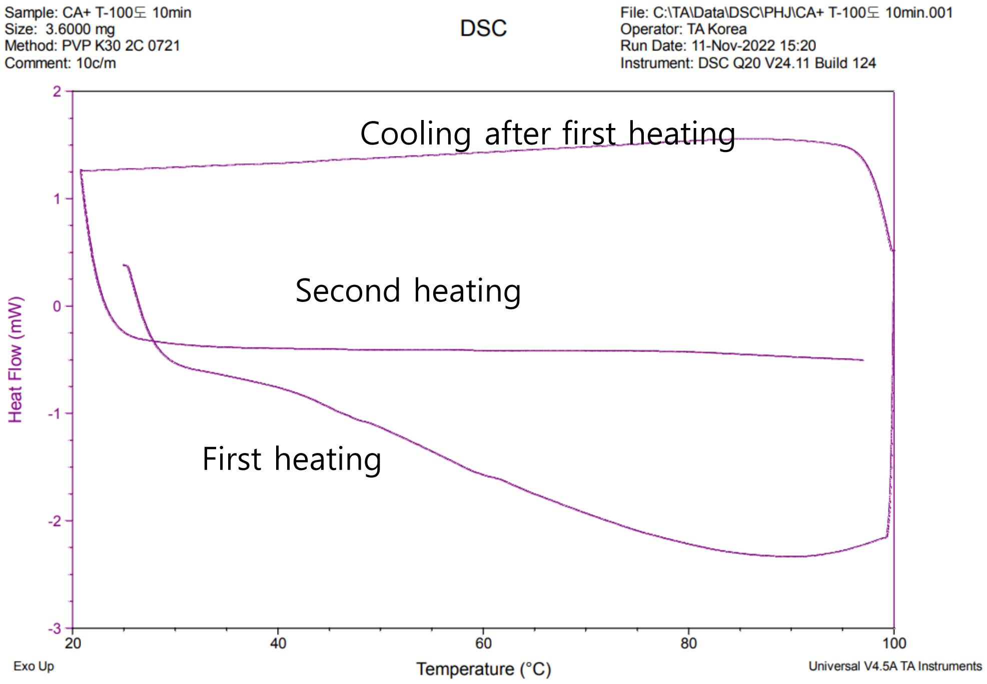

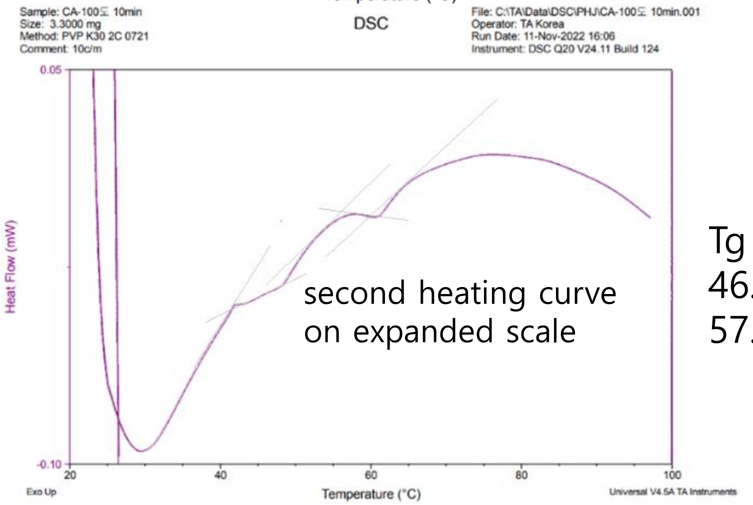

Tg
46.7 ℃
57.5 ℃

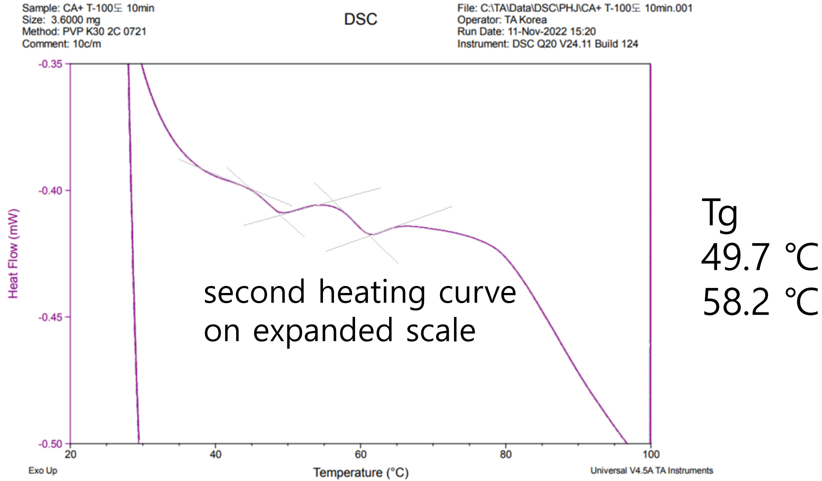

Tg
49.7 ℃
58.2 ℃
